# Long- and short-term pollution effect in megapolis assessed from magnetic and geochemical measurements on soils, tree trunk bark, and air filters

**DOI:** 10.1007/s10661-024-13194-w

**Published:** 2024-10-10

**Authors:** Kseniia M. Bondar, Iryna V. Tsiupa

**Affiliations:** 1 Institute of Geophysics, Polish Academy of Sciences, Ksiecia Janusza 64, 01-452 Warsaw, Poland; 2 Taras Shevchenko National University of Kyiv, 90 Vasylkivska Str, Kiev, 03022 Ukraine

**Keywords:** Magnetic susceptibility, Heavy metals, Soil, Tree trunk bark, Air filter, Megapolis, Kyiv

## Abstract

This study identifies factors influencing spatial and temporal variations in magnetic susceptibility and heavy metal content in soils and airborne particulate matter within the Kyiv megapolis, Ukraine, and highlights how source apportionment differs in the long and short run. Topsoil magnetic susceptibility anomalies of > 70 × 10^−8^ m^3^kg^−1^ are observed around old factories. The tree bark magnetic susceptibility map provides a record of industry general low emissions for the last 2–3 decades. The patterns of both spatial distributions confirm that factory emissions dominate the composition of particulate falling on the ground in urban area, with exclusion of streets with heavy traffic. Enhanced concentrations of Cu, Ni, and Zn have been found in urban soils, showing a positive correlation with magnetic susceptibility. Re-suspended road dust dominates temporal variation of particulate matter magnetic susceptibility collected on air filters. The air at busy streets is cleaner in winter, when the street dust gets immobilized by snow cover or freezing. Industries in Kyiv pose no significant effect on air quality; the concentrations of Cr, Ni, Cu, Zn, Cd, and Pb are at normal urban level with the exception of the near vicinity to factories. Air in streets with heavy traffic is enriched with Fe and Mn. Principal component analysis reveals different pattern of air pollution for the busy streets and yard areas. Yards are less affected by road dust; thus, contribution of industrial emissions can be distinguished. The results provide context for further quantification of any alterations in ecological state of Kyiv megapolis that may have arisen from socio-economic shocks and direct threats connected to the current war.

## Introduction

Environmental pollution is a big issue in many large cities around the world. It is a problem that impacts over a half of the global population, as according to the United Nations World Urbanization Prospects, more than 4.3 billion people live in urban areas (UN World Urbanization Prospects 2018; Ritchie et al., 2018, 2019). Topsoil and air basin in megapolises are known to be affected by various pollution sources, including residential, commercial, industrial, and construction activities, transportation, and power generation.

Particulate matter (PM) makes substantial portion of natural and anthropogenic aerosols produced and deposited in urban area. Either relatively coarse super-micrometric particles, mostly coming from construction sites, non-exhaust vehicle emissions, soil dust resuspension, and industrial fugitives, or fine sub-micrometric particles, mostly from combustion processes, forest fires, and the transformation of gaseous species, make up the mixture (Contini et al., 2014; Koukoulakis et al., 2019).

Urban PM is enriched with magnetite-like ferrimagnetic particles and thus shows enhanced magnetic properties (Hunt et al., 1984; Jele´nska et al., 2017). Close association of magnetic iron oxides with heavy metals in PM makes it a factor of serious health issues and mortality (Jordanova et al., 2012; Lequy et al., 2019). PM is accumulated in different collectors, such as soil, natural surfaces and biomonitors, spider webs, artificial surfaces, and air filters, providing possibility to quantitatively estimate its amount either on hourly/daily/monthly basis and in the long run applying magnetic method (Evans & Heller, 2003; Matzka & Maher, 1999; Hofman et al., 2017; Kapicka et al., 1999; Franciškovic-Bilinski et al., 2023; Muxworthy et al., 2001, 2003, 2022; Sagnotti et al., 2006; Fusaro et al., 2021; Jele´nska et al., 2017).

Magnetic susceptibility (k) mapping of the topsoil in industrialized, urban, forest, and rural areas was among the first and still mostly popular environmental magnetism research (Thompson & Oldfield, 1986, Petrovsky & Ellwood, 1999; Kapicka et al., 1999; Blundell et al., 2009; Frančišković-Bilinski et al., 2023; Bondar et al., 2024). Detailed k mapping in megapolis is crucial for understanding the primary factors influencing its distribution and to determine the sources of anomalies. The proper mapping accuracy could be achieved by sampling points in a sufficient amount for further spatial interpolation (Blundell et al., 2009; Gołuchowska & Wróbel, 2018; Jordanova et al., 2014; Rachwał et al., 2017). The distribution of points could be either uniform or based on some assumption, e.g., about the proximity to the pollution source. Further development of this approach is realized in geostatistical modeling incorporating environmental covariates (Fabijańczyk et al., 2017, Zhou et al., 2022).

Hoffman et al. (1999a, b) claim soils and sediments in the roadsides are clearly influenced by anthropogenic emissions up to a distance of 5 m. These values of soils susceptibility obtained at the road are about 10 times larger than background values in areas. Similar conclusion had been drawn by Shi and Cioppa (2006) from their study of profiles crossing a busy highway. The rapidity of the return of susceptibility values to the county background value probably explains the fact that highway does not appear as a major anomaly on the magnetic susceptibility map of Windsor–Essex County in Canada.

The magnetic properties of tree trunk bark can be used as a proxy for pollution at different sites in the city over the several decades time span (Brignole et al., 2018; Kletetschka et al., 2003). As reported by Van Mensel et al. (2023), tree trunk bark saturation remanent magnetization increased with increasing tree trunk circumference, probably via its relationship with tree age. Tree bark reflects the concentration of pollutants in the atmosphere, even though these pollutants are present in very low concentration in the ambient air and are not recognized as enhanced substance by air quality monitoring facilities (Hofman et al., 2017).

Characterization of air filters that capture airborne particulate matter is particularly relevant in megapolises (Dai et al., 2020; Hofman et al., 2017). Magnetic measurements on air filters are a reliable tool to estimate atmospheric pollution level (Sagnotti et al., 2006; Winkler et al., 2021; Muxworthy et al., 2022; Górka-Kostrubiec et al., 2024). Air quality monitoring programs in many countries involve heavy metal (HM) determinations in PM (Duan & Tan, 2013; Chen et al., 2022; Rodney et al., 2005; EEA, 2024). Concentrations of HMs such as Hg, Pb, V, As, Mn, Ni, Cr, and Cd found in the dust collected on air filters are regulated by the World Health Organization quality guidelines and estimated reference levels published in 2021 (WHO, 2021) and the EU Air Quality (Directive 2008/50/EC). By magnetic and geochemical measurements on air filters, it is possible to evaluate the influence of transportation emissions, combustion of fossil fuels, and various metallurgy and metal processing industries. Coal combustion results in a higher amount of harmful substances including particulates being emitted to the atmosphere comparing to other natural fuel combustion (Finkelman & Greb, 2008). In the cities, road dust represents serious environmental threat as a secondary source of air pollution caused by dust re-suspension (Jordanova et al., 2014). Time-related variations of the magnetic signature of PM are reported to be controlled by human activity, meteorological conditions, and natural dust flux (Sagnotti et al., 2006; Muxworthy et al., 2001, 2003).

The aim of this study was to evaluate environmental pollution level in Kyiv, the capital city of Ukraine, at the long-term retrospective (using topsoil magnetic susceptibility and heavy metals concentrations determinations), in the time span of last 3–5 decades (using magnetic susceptibility measurements of tree trunk bark) and at short-term span (i.e., using magnetic susceptibility and HM’s contents measured on air filters collected monthly), in relation with spatial distribution of pollution sources and types of urban locations. Therefore, the soils were sampled at 965 points; tree trunk bark from three tree species was sampled in 653 points and analyzed magnetically for the mass-specific susceptibility (χ). Interpolated maps of susceptibility aid identification of magnetic PM emitting industries. Determination of Mn, Ni, Cu, Zn, Cd, and Pb concentration levels on 225 soil samples was used to perform source apportionment using principal component analysis. Magnetic susceptibility measurements and determination of concentrations of heavy metals (Cr, Fe, Mn, Ni, Cu, Zn, Cd, Pb) were performed to characterize the anthropogenic PM collected during a period of 3.5 years on air filters at seven air sampling stations. Time variations provided valuable insight into PM quantitative and qualitative variation dynamics and uncovered common factors influencing air quality in the city. We assessed the spreading of anthropogenic PM over the on-ground ambient air in the city and investigated statistical links inside the dataset of magnetic susceptibility and content of HMs.

This study will serve as a baseline in future studies investigating changes in pollution rates particularly resulting from the war situation that began in 2022.

## Materials and methods

### Study area

**Fig. 1:**
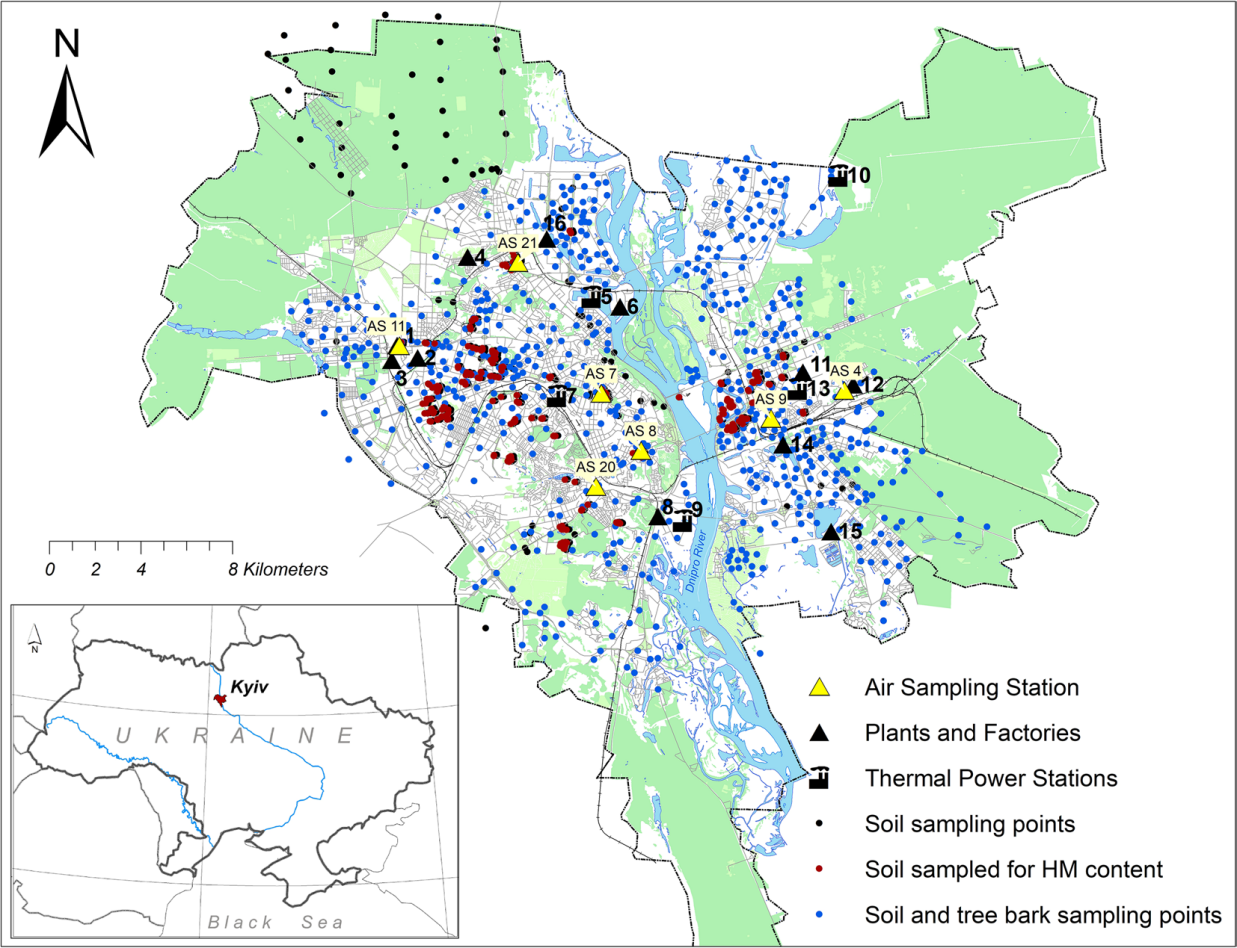
Map of the Kyiv city showing location of industries, soil and tree bark sampling sites, and air sampling stations. Industries shown on the map: 1, aviation plant; 2, machine tool factory; 3, machinery plant (closed); 4, electrical equipment factory; 5, TPS-2; 6, metalworking and ship construction plant; 7, TPS-1; 8, asphalt and concrete factory; 9, TPS-5; 10, TPS-6; 11, chemical factory (closed); 12, railway car repair factory; 13, Darnytska TPS; 14, ash landfill of Darnytska TPS; 15, municipal waste incineration plant; 16, concrete plant

Kyiv megapolis (N 50.45°; E 30.53°), the capital city of Ukraine, ranks among the ten largest cities in Europe in terms of both population and area. The total population in 2022 was 2,952,301 (Ecological passport of Kyiv in 2022, 2023). The city of Kyiv occupies an area of 835.6 km2 on both banks of the Dnipro River (Fig. 1). The natural conditions in Kyiv are influenced by its location at the boundary between two geographical zones, namely the Forest and the Forest-Steppe. The climate is temperate with warm summers, mild winters, and optimal humidity. The average annual air temperature is + 8.9– + 11.9 ℃. An important feature of microclimate is the presence of temperature contrasts between the flat left bank and the hilly right bank. Climatic conditions are significantly influenced by the city itself—heat dissipation from heating lines, buildings, thermal power plants, etc. In this regard, the air temperature in the city is higher than in its surroundings. The average annual rainfall is 600–700 mm. The predominant direction of the wind in summer is west, and in winter, it is northwest (Vyshnievskyi et al., 2023).

The soil cover in the city is variable. According to the WRB-FAO (IUSS Working Group WRB, 2015) classification, the *Albeluvisols Umbric* soil formed mainly under coniferous forests is identified at the northern suburbs of the city, while the southern part is covered by *Luvisols Haplic* formed on loess deposits.

Built-up land of the city reaches 364.0 km^2^ or 43.5%, of which 115.0 km^2^ is under residential and public buildings. A large area is occupied by industrial facilities, 56.0 km^2^, and transport and communication facilities, 22.0 km^2^. Kyiv is a fairly green city, but the vast majority of green areas are concentrated in separate or remote areas—near rivers and on the outskirts of the city next to forests.

Kyiv has a powerful industrial complex of multi-sector enterprises, in some places with outdated energy-consuming and resource-consuming technologies without proper purification of emissions. The industrial complex of the city is represented by power plants, construction industry, machine-building, metal and woodworking industry, chemical-pharmaceutical, food, and other industries. In 2018, the industry emitted to the atmosphere 29.2 thousand tons of harmful substances (Regional report, 2018). The main pollutants of Kyiv’s atmospheric air are mobile sources, among which motor vehicles dominate. When determining the total volume of emissions from mobile sources of pollution, emissions from railway, aviation, and water transport, production equipment are also included. The contribution of mobile sources is more than 70% of the total amount of pollutants in the ambient air of the city. In 2018, the mobile sources emitted to the atmosphere 142.5 thousand tons of harmful substances (Regional report, 2018).

The monitoring of the ecological state of the environment in Kyiv is conducted by the Central Geophysical Observatory named after Borys Srieznievsky (CGO). The air quality monitoring network set up by CGO consists of 21 sites; in seven of which, air sampling is performed. These seven are located as shown in Fig. 1 in different positions according to emission sources. This information is summarized in Table 1.

**Table 1:** Characterization of air sampling stations locations in Kyiv

Air sampling station	Address	Position characterization
AS4	2 Engineer Borodin str	At the eastern outskirt of the city, in the yard area between 5-storey buildings, ⁓75 m away from a road with tram tracks and ⁓200 m away from Darnytsia railway junction and car repair facility
AS7	Bessarabska Square	At the city center, in the square with heavy traffic and frequent traffic jams
AS8	29 Lesi Ukrainki av	At the city center, in the yard area between nine-story buildings, in 50 m from a busy crossroad bridge
AS9	10A Kaunaska str	At the left bank urban and industrial area, near the moderately busy road, in the direct exposure
AS11	98/2 Beresteiskyi av	At the avenue with heavy traffic and frequent traffic jams, just next to the aviation plant
AS20	Demiivska Square	At the city center, in the square with heavy traffic
AS21	5 Semena Sklyarenka str	In the yard, near the street with moderate traffic in the urbo-industrial area with enterprises of chemical industry and concrete production

### Sampling methodology and period

Soils were sampled in 2016–2018 years from the surface in 965 sites at undisturbed grassland distant from the road at least on 5 m to avoid spots heavily polluted with re-suspended road dust near busy streets and highways (Hoffmann et al., 1999a, b). Each sampling site represents a spot of about 4 m^2^, where material was taken from 5 points by “envelope” scheme and mixed. Sampling strategy aimed to cover evenly the whole city. Sampling sites were recorded using a hand-held GPS unit. Samples were air dried and grinded into a powder in a mortar.

Soil samples from 225 sites were selected to measure concentrations of HMs based on magnetic susceptibility map, reflecting their proximity to potential sources of heavy metal contamination, such as industrial areas and streets with heavy traffic. Residential area sites moderately affected by anthropogenic activities were also included.

Tree trunk bark samples were collected at the same locations as soils from trees of *Tília cordata*, *Ácer platanoides*, and *Aesculus hippocastanum* in 653 sites. These tree species were chosen as the most common ones in the city with furrowed bark texture, being a good trap for dust (Brignole et al., 2018; Van Mensel et al., 2023). Trees with a trunk diameter of 1.0–1.5 m were chosen, assuming the age of the tree to be 20–30 years. Each sampling point represents one tree growing at a distance of not less than 5 m from busy road or not less than 3 m from smaller road to avoid severe influence of traffic emissions (Hoffmann et al., 1999a, b; Shi & Cioppa, 2006). 0.5-mm-thick flakes of tree bark were taken at a height of 1.5 m from a “belt” encircling the tree trunk, using a porcelain knife. Before measurements, bark samples were dried at 100 ℃ in the muffle and grinded into a powder.

Sampling period of air filters spans from June 2015 till December 2018 in every air sampling site (AS) except AS-8, which had been installed in January 2016 and used since that time. Every month, 4–5 air filters were collected from each AS. The air monitoring stations pump an air volume of 30 m^3^ through each filter for 3–4 days (5–8 h a day). The pumping rate is approx. 0.02 m^3^ of air per minute. The PM is retained in a filter of AФA-XA-20 type with the area of 20 cm^2^. Fiber filter material ensures retention of more than 99.9% of particulate matter with aerodynamic diameter more than 0.1 µm. After the sampling is completed, the filter is rolled up with the working surface inward, placed in a paper bag, sealed, and stored in the refrigerator until processing.

### Magnetic susceptibility measurements of soils and tree bark

Volume magnetic susceptibility k was measured on samples packed into inch-size plastic boxes with the Bartington MS2B dual frequency meter (*k*_*LF*_ at 0.47 and *k*_*HF*_ at 4.7 kHz) with a measuring accuracy of 10^−5^ SI. The mass-specific magnetic susceptibility of soil (χ_S_) or tree bark (χ_B_) was calculated using the dry weight of the sample.

For soils, frequency dependence of magnetic susceptibility (k_fd_) was used as a proxy of relative contribution of the superparamagnetic (SP) fraction to the total magnetic susceptibility signal (Dearing et al., 1996). It is defined as:

1 $${k}_{fd}=\frac{({k}_{LF}-{k}_{HF})}{{k}_{LF}}*100\%$$

### Magnetic susceptibility measurements of filters

The magnetic susceptibility of air filters was measured in the Institute of Geology of Taras Shevchenko University of Kyiv, using a KLY-2 Kappabridge (Geofizyka, Brno). The instrument provides applied magnetic field of 300 A/m, operating frequency of 0.92 kHz, and sensitivity of 4 × 10^−8^ SI.

Three repeated measurements were done on monthly pack of filters (3–4 filters at once) placed together inside the pickup coil without plastic holders or boxes. The average values are corrected for susceptibility of unexposed filters. The magnetic susceptibility normalized per unit volume of air pumped through the filters during their exposure (k_V_) is defined as recommended by Górka-Kostrubiec et al. (2024). Thus, the result is monthly magnetic susceptibility of PM in air expressed in m^−3^. The reason of using kV instead of mass-normalized magnetic susceptibility is that PM accumulated on filters may contain diamagnetic and paramagnetic materials originating predominantly from the topsoil. While these materials contribute to the total mass of PM, they do not significantly affect k. As a result, normalizing k by mass may lead to an underestimation of anthropogenic (ferrimagnetic) contribution.

### Heavy metal determinations on soils

Concentrations of heavy metals (Mn, Ni, Cu, Zn, Cd, Pb) in soil samples were determined in the CGO with an atomic absorption spectrometry (AAS) method using spectrophotometer KAS-115 Saturn (JSC Selmi, Sumy, Ukraine). The detailed description of the sample preparation and measuring procedure is provided in Bondar et al. (2024).

### Heavy metals determinations on filters

Determination of concentrations of heavy metals (Cr, Fe, Mn, Ni, Cu, Zn, Cd, Pb) in PM gathered on air filters was carried out in the CGO with AAS method using spectrophotometer KAS-115 Saturn (JSC Selmi, Sumy, Ukraine) according to State Sanitary Rules for the Protection of Atmospheric Air in Populated Areas ДCП-201–97 (ДCП-201–97, 1997). The heavy metal content was determined in sample solutions in an air-acetylene flame. In order to obtain the solution, AФA-XA-20 filter is placed in a quartz beaker, and 5.0 cm^3^ of concentrated nitric acid was added. The beaker is heated until the emission of violent vapors stops. After cooling, 0.3 cm^3^ of hydrogen peroxide is added and kept for 30 min at room temperature. The solution is then heated and evaporated to wet salts. 0.2 cm^3^ of concentrated nitric acid is added to the residue transferred to the test tube. The volume of the solution in the test tube is made up to 5.0 cm^3^ by adding deionized water. The resulting solution is analyzed. The values obtained for unexposed filters are subtracted from the analysis results.

### Data analysis and statistical calculations

The Tomlinson pollution load index (PLI) (Angulo, 1996; Tomlinson et al., 1980) was used with the purpose to evaluate a level of heavy metal pollution in urban topsoil. We used five metals (Mn, Ni, Cu, Zn, Pb) to calculate the PLI. The PLI index is defined as the nth root of the multiplication of the concentration factors (CF_HM_), where CF_HM_ is the ratio between the concentrations of each heavy metal (C_HM_) to its corresponding background concentration (C_B_):

2 $$C{F}_{HM}=\frac{{\text{C}}_{HM}}{{\text{C}}_{B}}$$

3 $$PLI=\sqrt[n]{{CF}_{HM1}*{CF}_{HM2}*\dots *{CF}_{HMn}}$$

C_B_ refers to the HM contents in natural *Haplic Luvisols* of the Forest-Steppe of Ukraine (Fateiev & Pashchenko, 2003). Based on PLI, the sites can be identified as not polluted (PLI < 1), moderately polluted (1 < PLI < 2), heavily polluted (2 < PLI < 3), and extremely heavily polluted (PLI > 3) (Liu et al., 2013; Rachwał et al., 2017).

We used statistical software package IBM-SPSS to estimate the correlations and identify pollution sources by principal component analysis (PCA) within the datasets of magnetic parameters and heavy metal concentration in soils and air filters.

PCA was used as a mathematical procedure that extracts variables from the original data and groups them into factors known as principal components (PC) (Jolliffe, 2002). While remaining independent of one another, PCs attempt to capture as much variance in the data as possible. Two tests—the Bartlett’s test of sphericity and the Kaiser–Meyer–Olkin measure of sampling adequacy (KMO)—were used to infer the suitability of the data. KMO values greater than 0.5 suggest an appropriate degree of partial correlation between the variables. It can be determined using the Bartlett’s test of sphericity (Bartlett, 1951) whether a correlation matrix differs significantly from an identity matrix. For a correlation matrix to be appropriate for factor analysis, a significance less than 0.05 is required (IBM, 2021).

## Results and discussion

### Topsoil and tree bark magnetic susceptibility maps

**Fig. 2:**
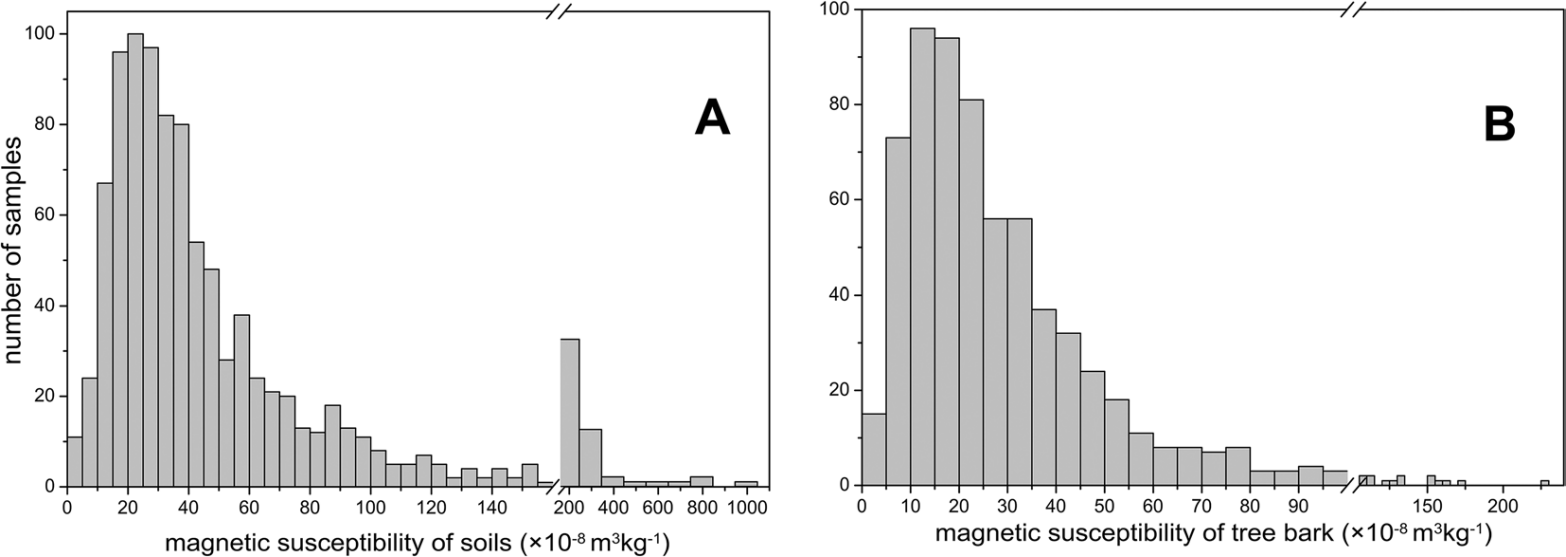
Histograms of magnetic susceptibility for the soil (**A**) and tree bark (**B**)

Mass-specific magnetic susceptibility of the topsoil χ_S_ in Kyiv varies between 1.4 and 960.6 × 10^−8^ m^3^kg^−1^ with a median value of 34.7 × 10^−8^ m^3^kg^−1^ (Table 2). Magnetic susceptibility of tree trunk bark χ_B_ also shows a large variability between 2.3 and 226.8 × 10^−8^ m^3^kg^−1^ with a median value of 22.9 × 10^−8^ m^3^kg^−1^ (Table 2). The large variability is typical for urban areas containing multiple sources of magnetic pollution (Magiera et al., 2007). The statistical distributions of both χ_S_ and χ_B_ are positively skewed due to a large number of samples from parks and forest suburbs as well as from clean residential areas, where magnetic susceptibility is close to a background value of 35 × 10^−8^ m^3^kg^−1^ determined for non-polluted *Luvisols Haplic* in this study (Fig. 2).

**Table 2:** Statistical parameters of soil and tree bark magnetic susceptibility

	Number of samples	Range	Mean ± SD	Median	Skewness	Coefficient of variation
χ_S_ (10^−8^ m^3^kg^−1^)	965	1.4…960.6	53.4 ± 70.6	34.7	6.4	1.3
k_fd_ (%)	847	0.0…13.1	3.4 ± 2.4	2.7	0.9	0.7
χ_B_ (10^−8^ m^3^kg^−1^)	653	2.3…226.8	30.1 ± 25.5	22.9	2.7	0.8

*χ*_*S*_ mass-specific magnetic susceptibility of soil, *k*_*fd*_ frequency dependence of magnetic susceptibility of soil, *χ*_*B*_ mass-specific magnetic susceptibility of tree trunk bark, *SD* standard deviation.

**Fig. 3:**
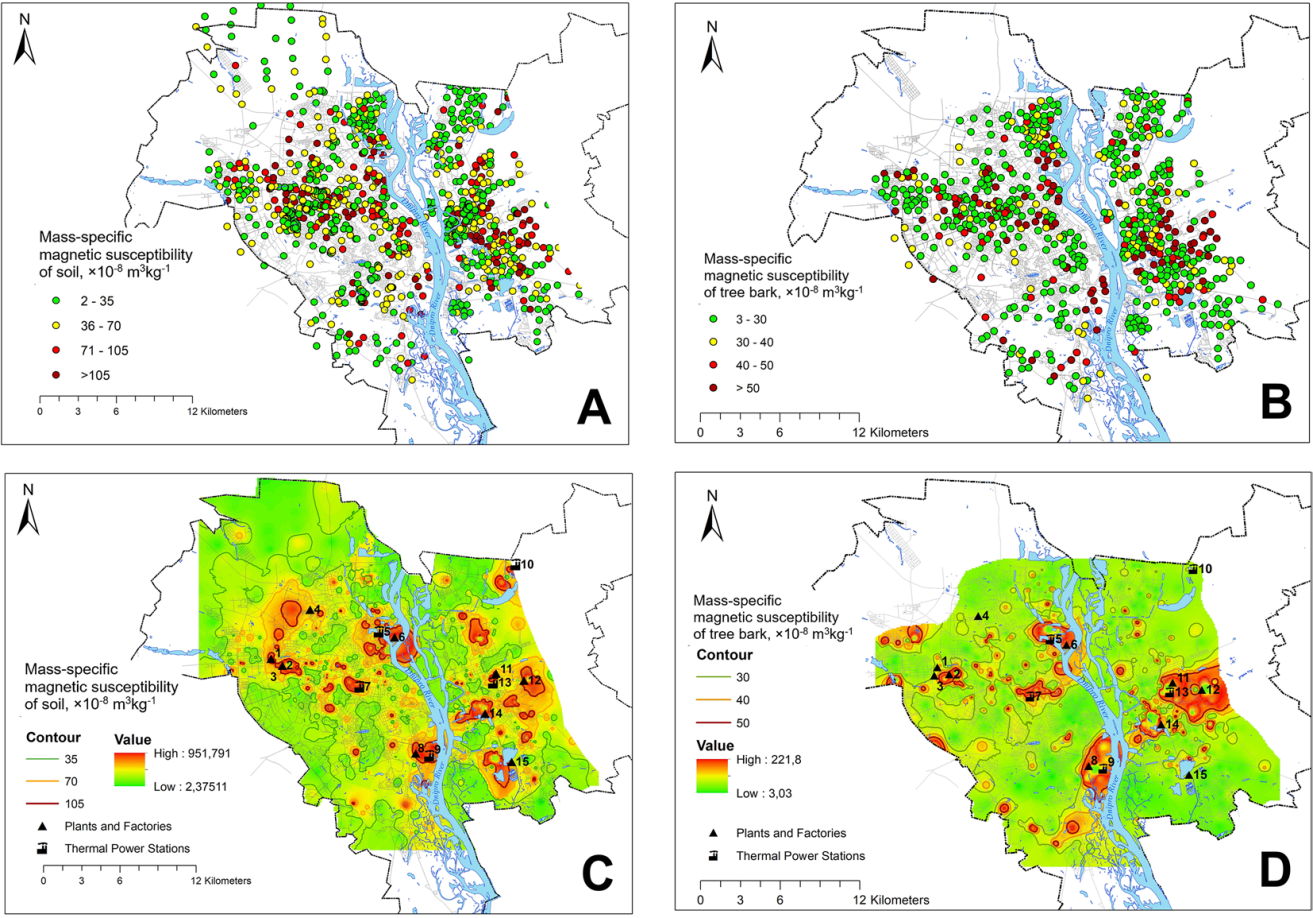
Magnetic susceptibility maps of Kyiv: post-classed map of measured soils χ_S_ (**A**); post-classed map of measured tree bark χ_B_ (**B**), IDW interpolated χ_S_ (**C**); IDW interpolated χ_B_ (**D**)

The spatial distribution of χ_S_ and χ_B_ is simulated using ArcGIS Spatial Analyst tool. Figure 3A and B illustrates distribution of measured χ_S_ and χ_B_ on sampling sites and Fig. 3C and D shows maps created using inverse distance weighted (IDW) interpolation method, which is at the same time exact and deterministic, when it is applied to pollution problems (Mesnard, 2013).

**Fig. 4:**
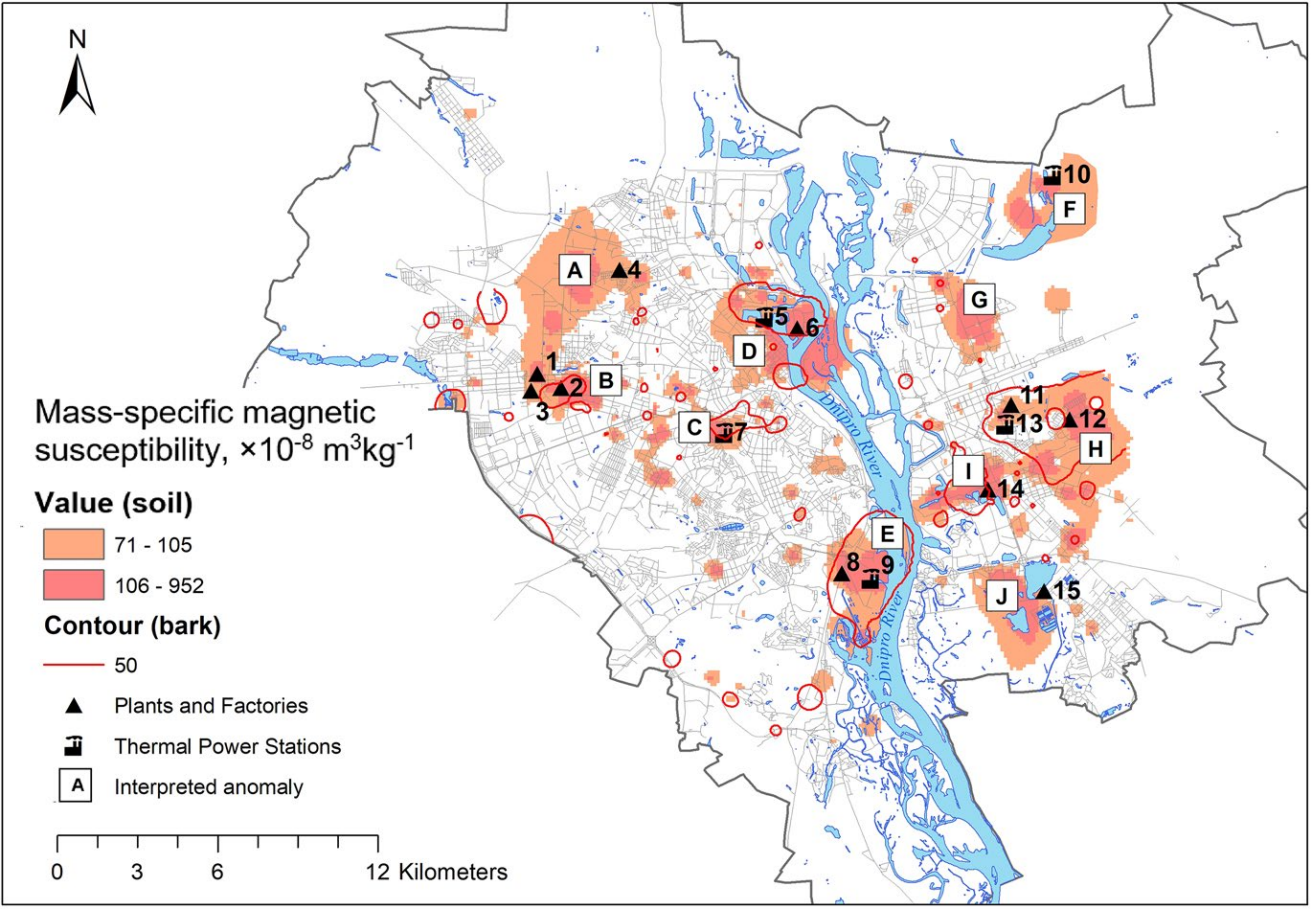
Geographical distribution of magnetic susceptibility χ_S_ and χ_B_ anomalies in relation to polluting industries. Industries shown on the map: 1, aviation plant; 2, machine tool factory; 3, mechanical engineering plant (closed); 4, electrical equipment factory; 5, TPS-2; 6, metalworking and ship construction plant; 7, TPS-1; 8, asphalt and concrete factory; 9, TPS-5; 10, TPS-6; 11, chemical factory (closed); 12, railway car repair factory; 13, Darnytska TPS; 14, ash landfill of Darnytska TPS; 15, municipal waste incineration plant

The patterns of χ_S_ and χ_B_ maps show similarities and differences, delineating areas of major technogenic impact ongoing and in the past (Fig. 4). Anomalies B, C, D, E, H, and I with highest values (χ_S_ > 70 × 10^−8^ m^3^kg^−1^ and χ_B_ > 50 × 10^−8^ m^3^kg^−1^) are clearly detected as spots around their source. Anomaly B covers the area of two industries: machine tool factory operating since 1933 until recently and mechanical engineering plant existing in 1898–2012 (Ishchuk & Gladkij, 2005). To the north of it, we can see topsoil χ_S_ anomaly A at the location of aviation plant and airfield. Anomalies C and E occupy areas nearby TPS-3 and TPS-5, respectively. Anomaly D was measured in the northern part of the city at the location of one of the oldest industries—steel production (since 1862), machine-building, metalworking and ship construction (since 1928) plant, and adjacent thermal power station TPS-2, which had been operating since 1930 and used mainly coal fuel, since the 1980s had been switched to mazut and natural gas. In the last decades, this industry junction significantly reduced production volumes, which resulted in less emissions and smaller area of χ_B_ anomaly.

At the left-bank city, anomaly H has been formed at the place of actively operating Darnytsia industry junction with fossil fuel combustion Darnytska TPS, large chemical plant (closed by now), the railway repair plant, and industrial railway station. As it could be assumed from the χ_S_ and χ_B_ maps, the railway transportation facilities cause significant magnetic pollution. Anomaly H originates from dusting of ash and slag waste landfill of Darnytska TPS. The landfill is organized in natural lake, which, however, was used as a technical pond for almost 60 years. According to satellite monitoring data, carried out from 2005 to 2021 by the National Space Facilities Control and Test Center, the area of the water surface is rapidly decreasing: in 2005, it was 8.58 ha, and in 2020, it was only 0.98 ha (Specialists of the National Space Facilities Control and Test Center…, 2021). Emissions are delivered constantly and slowly settle to the bottom; as of today, the water surface is almost absent.

At the territory of the city, we can observe several topsoil anomalies which are not accompanied with tree bark anomalies. These are A, F, G, and J. Anomaly A occupies large area at the northern west of the city. Its origin is not clear enough. It covers locality around aviation plant and adjacent airfield and electrical equipment plant. Possible explanation could be wildfires that affected the territory in the past and promoted formation of magnetic iron oxides only within soil (Jordanova et al., 2018). Anomaly F is formed in the radial proximity of 2 km to TPS-6. Anomaly G being located at recent residential locality delineates an area of former artillery shooting range (Kyiv: encyclopedic guide, 1981); it could have the same origin as anomaly A or could have formed due to admixture of iron ammunition residue (Tauqeer et al., 2021). Anomaly J is presumably formed due to emissions of municipal waste incineration plant. Magnetic enhancement here could be observed on χ_S_ and less prominent on χ_B_ map.

According to maps represented on Fig. 3, the urban area generally demonstrates enhanced values of topsoil susceptibility comparing to unhabituated forest outskirts. Thus, at the city streets, the road dust particles that have been lifted into the air and settled on the ground contain ferromagnetic iron oxides in high abundance.

### Frequency dependence of magnetic susceptibility in soils

**Fig. 5:**
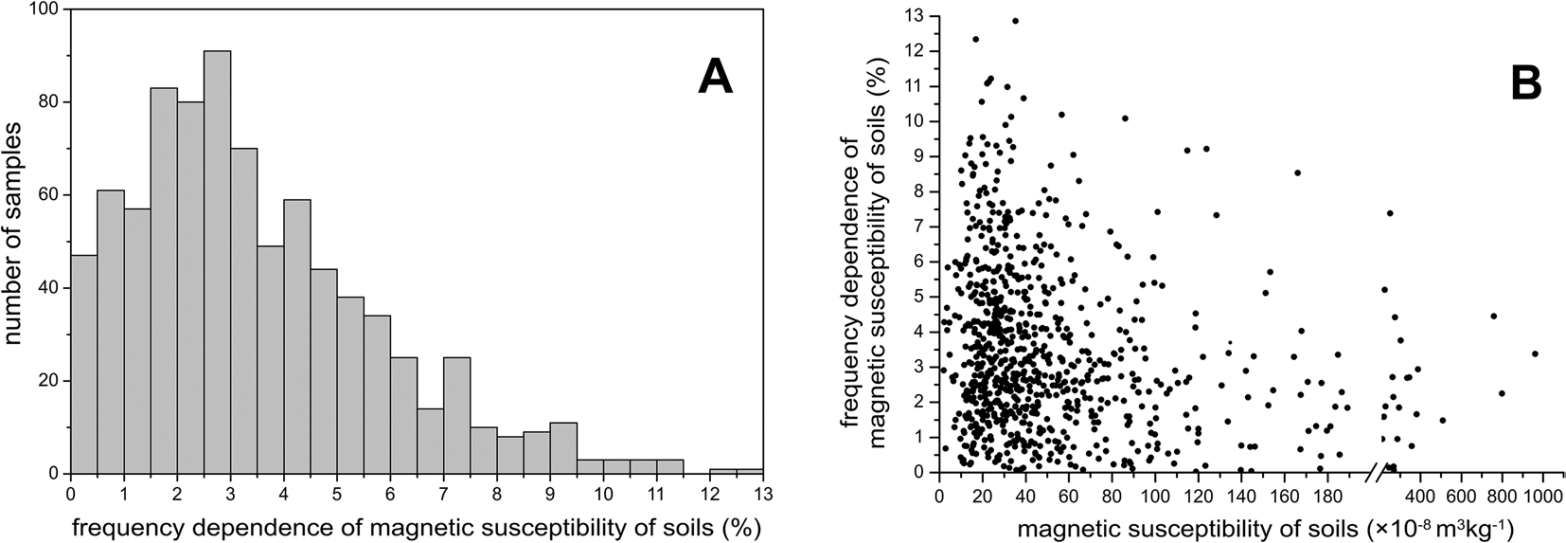
Histogram of frequency dependence of magnetic susceptibility k_fd_ (**A**) and its variation with change in magnetic susceptibility (**B**) of soils

Figure 5A represents a histogram of k_fd_ of soils. Values vary between 0 and 13.1%, with a mean of 3.4%. These results indicate the contribution from variable concentrations of ultrafine SP particles in the studied samples. The minimal values indicate the predominance of coarse particles, while maximal k_fd_ values signify that the concentration of the ultrafine particles is higher. Although ultrafine particles are recently found in anthropogenic emissions (Gonet et al., 2021b), their mass contribution to the urban soil is vanishingly low (Dytłow et al., 2019). High k_fd_ signal in soil is mostly related to ultrafine-grained ferrimagnetics of pedogenic origin (Górka-Kostrubiec et al., 2016). Generally, the urban soils show tendency of decrease in k_fd_ when the χ_S_ increases (Fig. 5B).

### Heavy metal concentrations in urban soil

Heavy metal contents were determined in soils sampled from locations shown on Fig. 1. The statistical characterization of heavy metal concentrations in soils is given in Table 3. Heavy metal concentrations in the topsoil decrease in the following order: Mn > Zn > Pb > Cu > Ni > Cd (Table 3). The pollution could be accessed against the background concentrations of HMs in natural soils. For the *Luvisols Haplic* from Kyiv region, the background values in mg/kg established by Fateiev and Pashchenko (1991) are the following: Pb, 10; Zn, 56; Mn, 758; Cu, 14.5; and Ni, 22.

**Table 3:** Descriptive statistics of magnetic properties and HM contents in urban soils (*n* = 225)

	Range	Mean ± SD	Median	Skewness	Coefficient of variation
χ_S_ (10^−8^ m^3^kg^1^)	4.6–295.1	35.1 ± 32.1	27.7	4.4	0.91
k_fd_ (%)	0.0–11.2	3.7 ± 2.5	3.3	0.6	0.66
Cd (mg/kg)	0.00–4.75	0.24 ± 0.41	0.25	6.6	1.73
Mn (mg/kg)	61–947	289.61 ± 137.58	264.0	1.6	0.48
Cu (mg/kg)	1–143	18.92 ± 20.98	12.0	3.3	1.11
Ni (mg/kg)	0–26	10.36 ± 4.13	10.0	0.6	0.40
Pb (mg/kg)	3–414	24.99 ± 40.06	14.0	5.9	1.60
Zn (mg/kg)	13–1186	162.10 ± 126.19	145.0	3.3	0.78

*χ*_*S*_ mass-specific magnetic susceptibility of soil, *k*_*fd*_ frequency dependence of magnetic susceptibility of soil, *SD* standard deviation.

Concentrations of Mn and Ni in most samples are lower than the background and especially the reference values of 2000 mg/kg for Mn (Ashraf et al., 2021) and 50 mg/kg for Ni (Tóth et al. 2016), established for these elements.

The mean contents of metals in studied soils exceed the background in 2.5 times for Pb and 3 times for Zn, indicating these elements as main pollutants. Soil also shows enhanced concentration of Cu.

The pollution load index calculated for the five metals (Mn, Ni, Cu, Zn, Pb) shows values below 1 for the 70% of samples indicating absence of pollution. Twenty-four percent of samples are considered moderately polluted (1 < PLI < 2) and 6% of samples demonstrate heavy pollution level (PLI > 2).

### Links between magnetic susceptibility and heavy metals in soils and relation to pollution sources

The statistical correlations between χ_S_ and HMs are based on the Spearman’s rank coefficients. They are generally low but, within the dataset of 225 samples, still reveal statistically significant positive correlations between χ_S_ and Ni, Cu, Zn, and PLI as well as negative links between k_fd_ and Zn (Table 4). Poor correlations suggest nonlinear relationships between metal concentrations and soil magnetic susceptibility (Dai et al., 2020); thus, we assume the presence of several sources of magnetic particles containing HMs. These sources can vary greatly by the contribution to general pollution level.

**Table 4:** Correlation matrix showing linkage between magnetic parameters, content of heavy metals, and PLI index for soil samples. Number of samples *n* = 225. R_s_ correlations significant at the 0.01 level are printed in bold type

	Cd	Mn	Cu	Ni	Pb	Zn	χ_S_	k_fd_	PLI
Cd	1								
Mn	0.108	1							
Cu	**0.376**	0.120	1						
Ni	**0.247**	**0.321**	**0.350**	1					
Pb	**0.298**	0.082	**0.659**	**0.258**	1				
Zn	0.135	0.066	**0.587**	0.077	**0.405**	1			
χ_S_	0.073	0.164	**0.208**	**0.177**	0.129	**0.185**	1		
k_fd_	− 0.062	0.125	− 0.147	0.091	− 0.107	− **0.252**	− **0.179**	1	
PLI	**0.382**	**0.314**	**0.881**	**0.491**	**0.774**	**0.688**	**0.249**	− 0.163	1

*χ*_*S*_ mass-specific magnetic susceptibility of soil, *k*_*fd*_ frequency dependence of magnetic susceptibility of soil, *PLI* pollution load index.

Principal component analysis has been applied as a source apportionment tool. The suitability of data for structure detection was established by KMO value of 0.673 and Bartlett’s test < 0.001. Based on Kaiser’s criterion (Kaiser, 1960), three main PCs were selected that describe the model by 58.662%. The values of communalities higher than 0.5 indicate that all variables are well explained by the model. The varimax rotation method was used to simplify the interpretation of PCs (Table 5).

**Table 5:** Components extracted by PCA factorization of the topsoil data characterized by their significant correlations with variables

	PC1 Eigenvalue 2.379 Explained variability 24.586%	PC2 Eigenvalue 1.236 Explained variability 18.958%	PC3 Eigenvalue 1.077 Explained variability 15.118%	Communality
Cd	0.663			0.491
Cu	0.822			0.738
Pb	0.648			0.510
Zn	0.629			0.577
Mn		0.717		0.519
Ni		0.761		0.620
χ_S_			0.568	0.567
k_fd_			− 0.789	0.671

*χ*_*S*_ mass-specific magnetic susceptibility of soil; *k*_*fd*_ frequency dependence of magnetic susceptibility of soil.

**Fig. 6:**
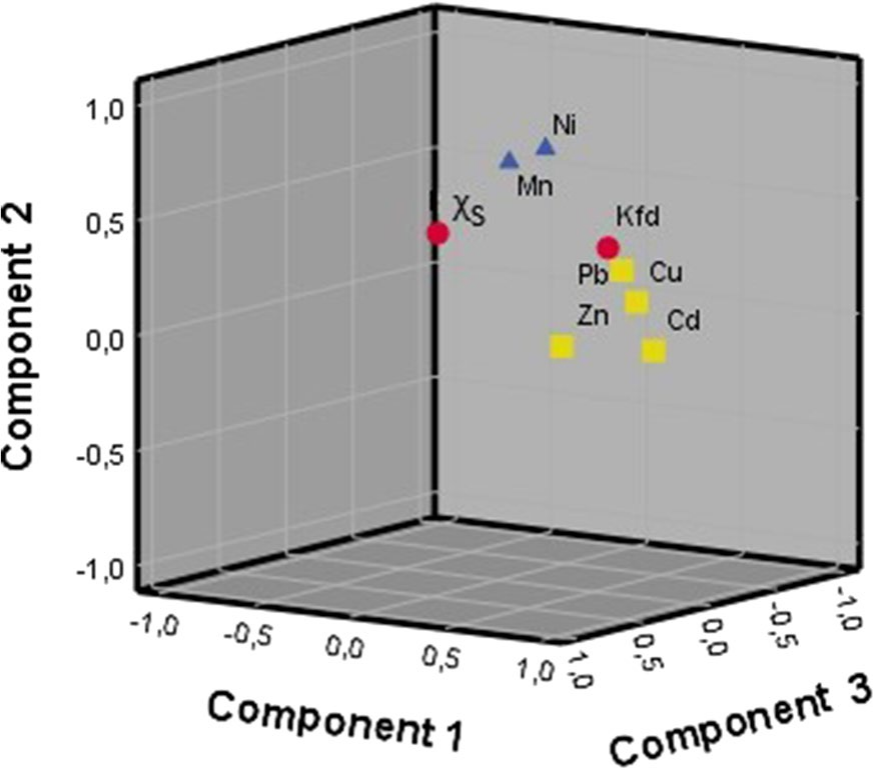
PCA results for the topsoil samples: 3D representation of loadings of the three principal components (circles, PC1; triangles, PC2; crosses, PC3)

The PC1 has the highest eigenvalue and explained variability (2.379 and 24.586%); it is significantly positively correlated with concentrations of Cu, Cd, Pb, and Zn suggesting that they have anthropogenic origins and share a common source, such as low emissions of long working industries including power plants. PC2 is strongly correlated with Mn and Ni having eigenvalue and explained variability of 1.236 and 18.958%, respectively. Concentrations of Mn and Ni in the urban soil are similar to natural ones and characterized by low coefficients of variation (Table 4). This is a reason to identify PC2 as representing natural pedogenic signal. PC3 has the lowest eigenvalue (1.077) and moderate correlations with χ_S_. At the same time, PC3 demonstrates negative correlation with k_fd_ and describes 15.118% of the total variance. This component represents the abundance of fine to coarse magnetic fraction such as airborne magnetite from the exhaust and non-exhaust sources of vehicular traffic deposited in street dust, which is anticorrelated to the presence of ultrafine particles that are mostly pedogenic (Bucko et al., 2011; Dytłow et al., 2019; Gonet et al., 2021a). Figure 6 shows the relationships between variables based on the three PCs.

### Spatial and temporal variation of magnetic susceptibility of PM accumulated on air filters

**Fig. 7:**
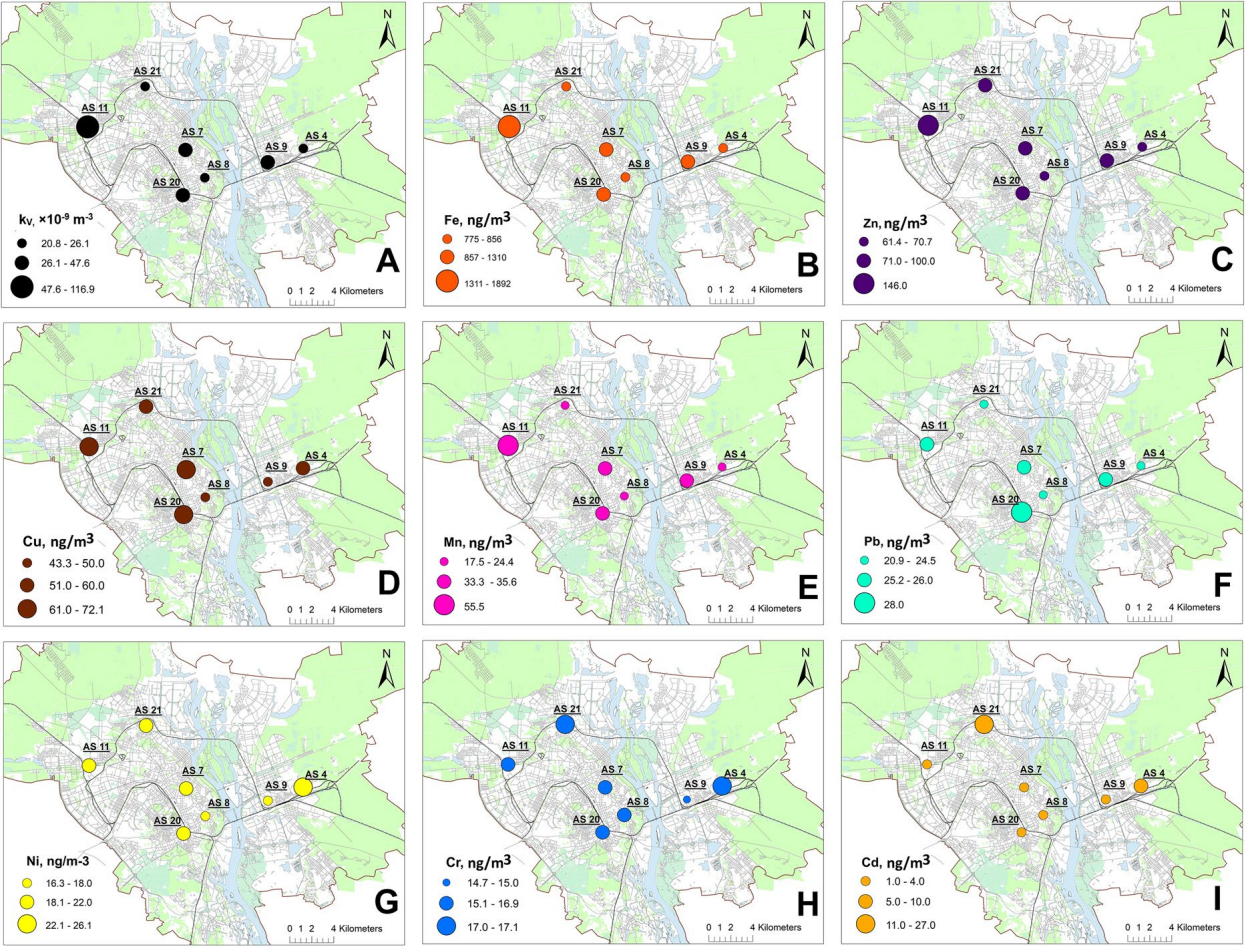
Spatial distribution maps of the values of k_V_ (**A**) and heavy metals content (**B**–**E**) averaged over a period of June 2015–December 2018 in the PM collected on air filters at air sampling sites in the city of Kyiv

Spatial variation of magnetic susceptibility k_V_ averaged over the period of observations for the AS stations is presented in Fig. 7A. The highest period averaged k_V_ is observed at the station AS11 (118 ± 42.0 × 10^−9^ m^−3^), while the lowest are at AS4 (23.3 ± 11.2 × 10^−9^ m^−3^) and AS8 (25.6 ± 11.9 × 10^−9^ m^−3^) (Table 6).

**Table 6:** Comparison of observation period mean magnetic susceptibility k_V_ (10^–9^ m^−3^) and selected HM`s concentrations (ng/m^3^) in air between air sampling sites in Kyiv

	Kyiv Mean ± SD							Maximum Permissible Concentrations of HMs (µg/m^3^) (ДCП-201–97, 1997)	Limit* and target** values for HMs in the PM of ambient air (ng/m^3^)
	AS4	AS7	AS8	AS9	AS11	AS20	AS21		
k_V_	23.3 ± 11.2	52.2 ± 18.3	25.6 ± 11.9	44.2 ± 20.1	118 ± 42.0	50.6 ± 18.6	30.5 ± 16.3	-	
Pb	24.5 ± 18.1	25.2 ± 16.1	20.9 ± 15.9	25.8 ± 18.8	26.0 ± 14.9	28.0 ± 33.7	24.0 ± 21.7	0.3	500 (*,**)
Cd	10.0 ± 6.65	3.09 ± 4.57	1.42 ± 1.93	2.28 ± 3.98	2.79 ± 4.18	4.02 ± 5.04	26.7 ± 19.2	0.3	5 (*,**)
Mn	24.4 ± 11.2	33.3 ± 16.1	17.5 ± 10.3	35.6 ± 16.5	55.0 ± 29.8	34.9 ± 21.0	22.3 ± 9.96	1.0	150 (**)
Ni	26.1 ± 29.2	20.1 ± 14.2	16.3 ± 9.43	16.7 ± 9.22	18.9 ± 11.2	19.1 ± 15.2	21.5 ± 11.3	1.0	20 (**); 25 (*)
Cr	17.0 ± 8.87	16.5 ± 11.2	15.5 ± 7.71	14.7 ± 8.75	15.4 ± 10.2	15.7 ± 13.3	17.1 ± 13.6	1.5	
Fe	846 ± 310	1310 ± 882	775 ± 366	1178 ± 526	1891 ± 973	1190 ± 866	855 ± 417	4.0	
Cu	55.4 ± 54.8	65.6 ± 27.8	43.3 ± 15.3	46.7 ± 22.4	72.1 ± 46.5	67.7 ± 46.4	54.0 ± 42.4	2.0	
Zn	70.7 ± 45.8	88.6 ± 34.6	61.4 ± 24.5	86.3 ± 48.9	146 ± 139	93.7 ± 59.1	93.5 ± 66.9	50.0	

*WHO (2021)

**Directive 2008/50/EC

**Fig. 8:**
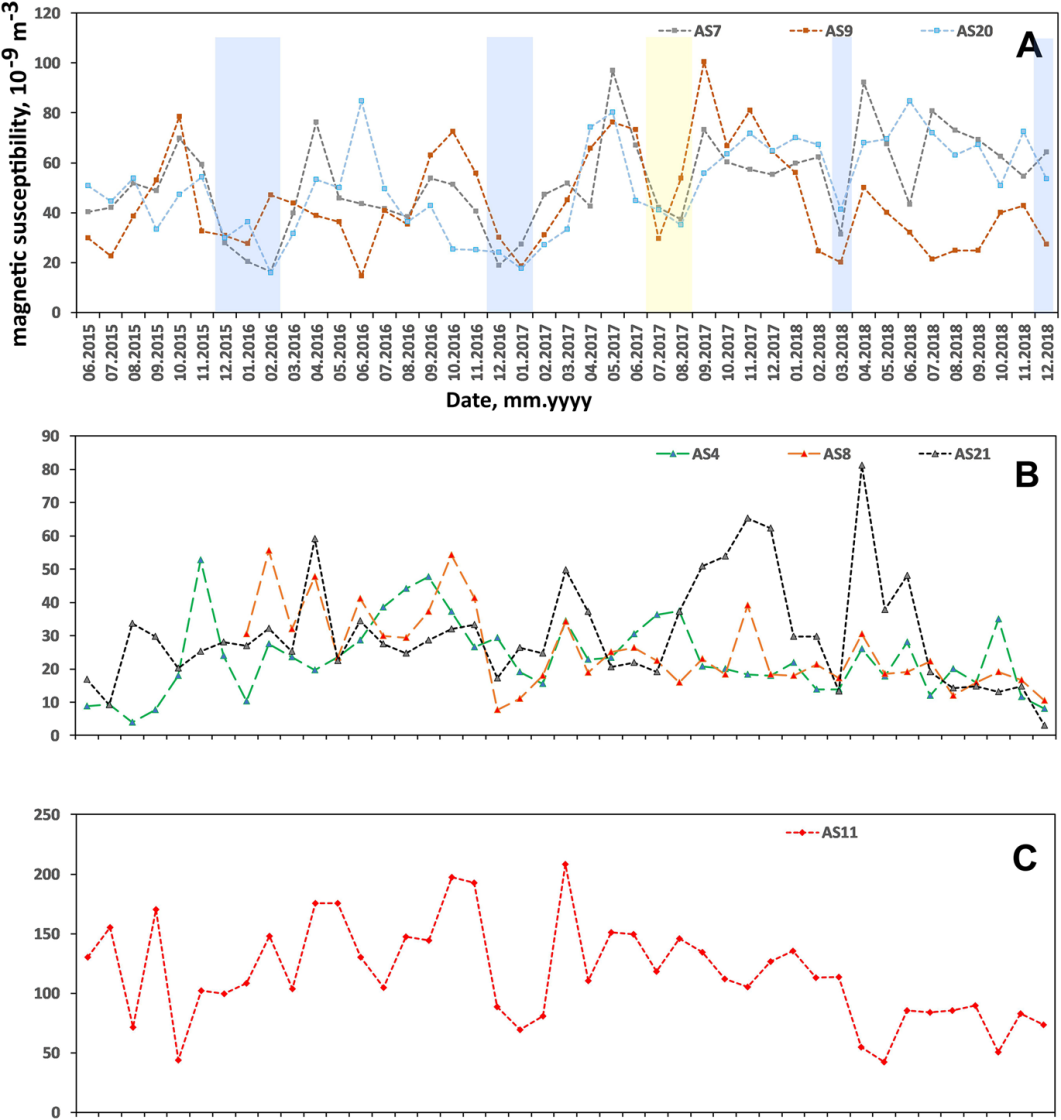
Temporal variation of magnetic susceptibility of PM collected on air filters at air sampling sites of cluster I (**A**), cluster II (**B**), cluster III (**C**). Snow cover duration periods are marked with blue stripes, and rainfall period of summer 2017 is marked with yellow stripe

Monthly k_V_ in Kyiv varies within the range of 3.02–211 × 10^−9^ m^−3^ (Table 7). Temporal variation patterns on different AS demonstrate visible correlations, however, being located far from one another in the city (Fig. 8).

**Table 7:** Summary on PM`s magnetic susceptibility k_V_ (10^−9^ m^−3^) and HM contents (ng/m^3^) in urban air

	Dataset for entire city (*n* = 294)		Cluster I (AS7, AS9, AS20) Heavy traffic area (*n* = 129)		Cluster II (AS4, AS8, AS21) Yard residential area (*n* = 122)	
	Range	Range	Range	Range	Range	Mean ± SD
k_V_	3.02–211	14.4–100	3.02–81.1	3.02–81.1	14.4–100	49.0 ± 19.2
Pb	0–220	0–220	0–120	0–120	0–220	26.4 ± 24.0
Cd	0–80	0–20	0–80	0–80	0–20	3.13 ± 4.57
Mn	10–120	10–120	10–70	10–70	10–120	34.6 ± 17.9
Ni	0–200	0–70	0–200	0–200	0–70	18.6 ± 13.1
Cr	0–70	0–70	0–70	0–70	0–70	15.7 ± 11.2
Fe	60–5170	60–5170	200–2520	200–2520	60–5170	1226 ± 772
Cu	0–360	0–310	10–360	10–360	0–310	60.0 ± 34.9
Zn	0–770	0–320	10–480	10–480	0–320	90.0 ± 48.3

**Fig. 9:**
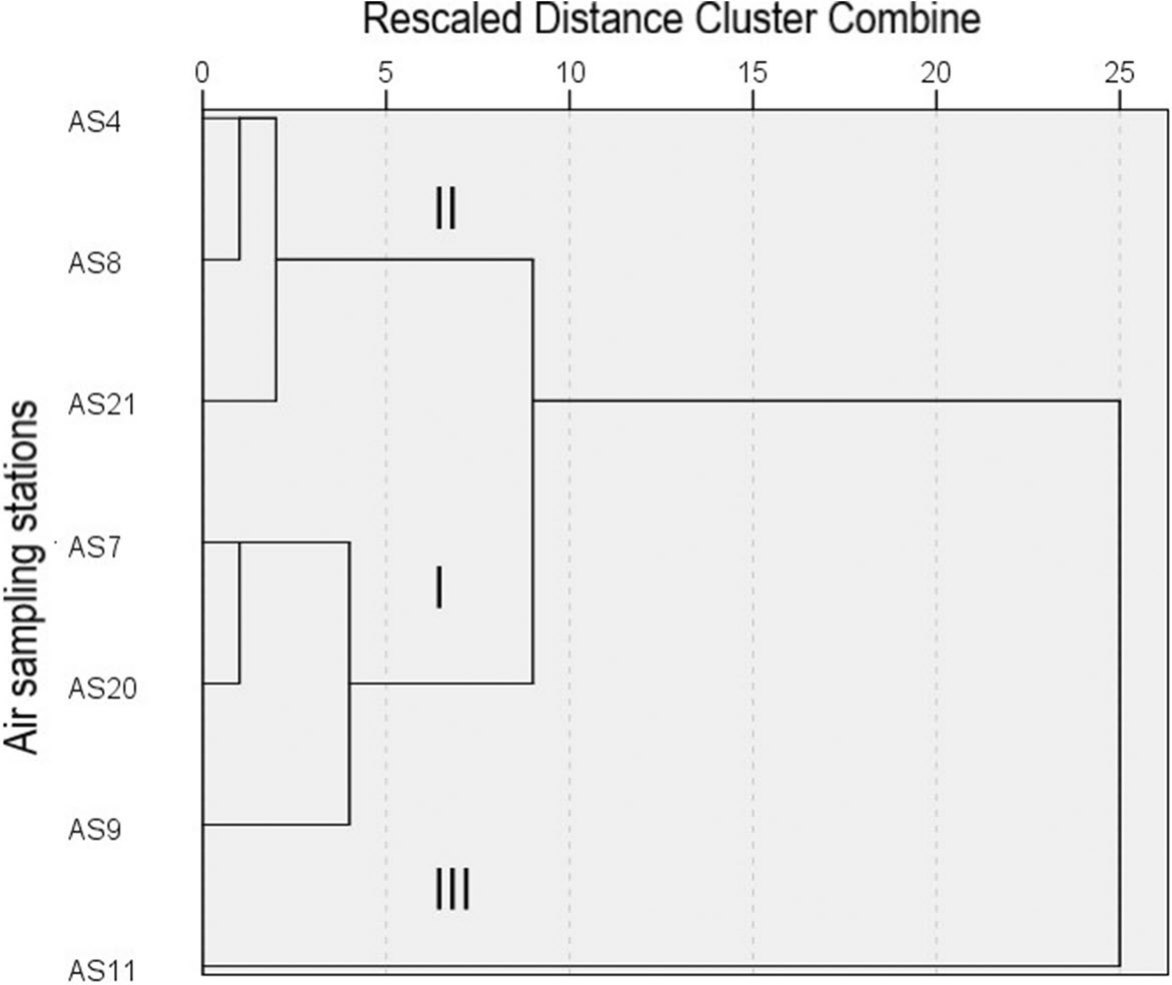
Cluster analysis results: dendrogram of air sampling sites classified by k_V_ temporal variation pattern

Cluster analysis has been performed to categorize the air sampling sites based in temporal variations of k_V_. The dendrogram shown in Fig. 9 illustrates division of dataset into three clusters with common conditions influencing PM accumulation dynamics in each one. Cluster I contains locations with higher long-period averaged k_V_ values AS7, AS9, and AS20 directly exposed to busy roads. Cluster II consists of sites AS4, AS8, and AS21 with low long-period averaged k_V_ values, located inside residential yard areas. Cluster III consists of the only site AS11 located next to the aviation plant which shows the highest k_V_.

Data from air sampling sites of cluster I (Fig. 8A) show minimums during winters of 2015–2016 and 2016–2017, in July–August 2017 and in March 2018. Winter minimums correspond to the snow cover duration (http://cgo-sreznevskyi.kyiv.ua/uk/), which are marked with blue stripes. Snow cover, frosty, or wet weather conditions immobilize the dust at the streets. In particular, March 2018 became the coldest and wettest in the twenty-first century, when 82 mm of precipitation fell as snow while the norm is 37. It followed the snowless winter. Summer minimum in 2017 is probably caused by rainfall, when dust particles which remain suspended in air get loaded and come down on the ground. Increase in k_V_ is observed in warm periods of the year and is connected to resuspension of dust by vehicles.

These seasonal variations are less visible or not observed for sites from cluster II (Fig. 8B), as they are protected from traffic exposure by buildings and plantings.

In cluster III represented by the only AS11, the k_V_ variations are controlled by industrial production on aviation plant. Here we observe high values from July 2015 till December 2017, probably related to activation of production (Fig. 8C).

### Heavy metal contents in PM accumulated on air filters

Heavy metal concentration in air particulate matter of Kyiv decreases in the following order: Fe > Zn > Cu > Mn > Pb > Ni > Cr > Cd (Tables 6 and 7). Generally, the contents of heavy metals in the atmospheric PM of Kyiv demonstrate moderate values never exceeding maximum permissible concentrations (Table 6, ДCП-201–97).

The observed HM concentrations strongly depend on the type of location. In the AS of cluster II (yard areas isolated from busy streets), Fe concentration lies in the range of 200–2520 ng/m^3^ with the mean value of 828 ± 366 ng/m^3^, while in near-road AS from cluster I, we observe mean value of 1226 ± 772 and a range of 60–5170 ng/m^3^. This is higher than reported for residential areas and even beyond the level of heavily industrialized sites. Koukoulakis et al. (2019) reported mean values of 741 ng/m^3^ in 2016 year and 869 ng/m^3^ in 2017 year measured in Elefsis industrial area in Greece. In New York City, it ranged from 52 to 418 ng/m^3^ in summer and from 4.364 to 488 ng/m^3^ in winter (Peltier & Lippmann, 2011). However, in residential areas of the steel producing city in Korea, Fe mean value was reported as 3400 ng/m^3^ (Chen et al., 2022).

**Fig. 10:**
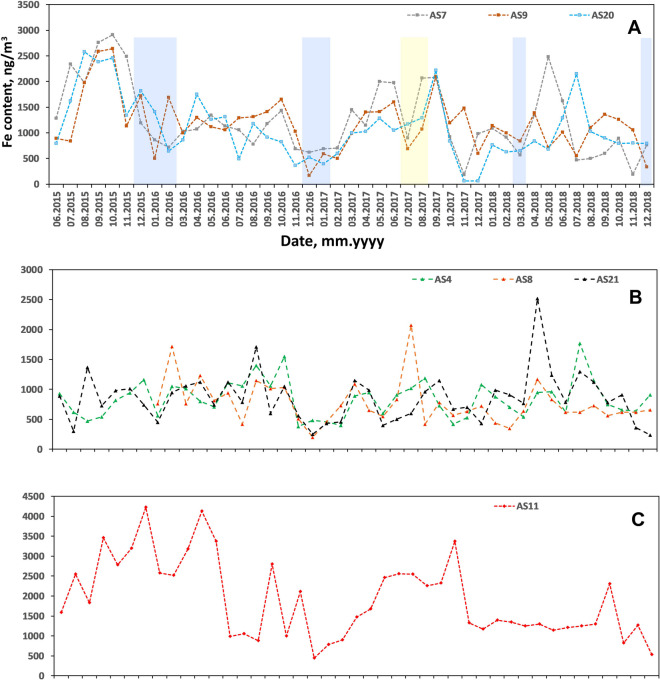
Temporal variation of Fe content in PM collected on air filters at air sampling sites of cluster I (**A**), cluster II (**B**), and cluster III (**C**). Snow cover duration periods are marked with blue stripes, and rainfall period of summer 2017 is marked with yellow stripe

Spatial distribution of Fe in urban air shows the same pattern as k_V_ (Fig. 7B). Temporal variations also are very similar to k_V_ (Fig. 10). On the curves from cluster I additionally to winter minimums, a clear maximum for August–October 2015 can be seen (Fig. 10A). This can be explained by additional soil dust flux caused by drier soil and stronger winds due to extremely hot summer (average temperature of summer 2015 was + 21.6℃, which is 2.0 ℃ higher than the climatic norm). The summer of 2015 took the 2nd place among the warmest after the summer of 2010 for the entire period of observations in Kyiv since 1881 (http://cgo-sreznevskyi.kyiv.ua/uk/). In addition, the summer of 2015 turned out to be the driest in the entire history of observations: only 30% of the precipitation of the summer norm, 68 mm, fell, and only 1.5 mm of precipitation fell in August.

Period averaged concentrations of Zn (92.1 ± 73.4 ng/m^3^) and Cu (58.1 ± 40.2 ng/m^3^) in the city of Kyiv are at common urban level, as for example, according to Rodney et al. (2005), in the USA, on average, Zn is less than 1 mg/m^3^ and the mean Cu concentrations range between 5 and 200 ng/m^3^ in rural and urban atmospheres (Dorsey et al., 2004) (Fig. 7C, D). Zn is higher in AS form clusters I and III (AS11), while Cu is enhanced at AS7, AS11, AS20, and AS21 indicating origin of these elements from industrial and vehicular emissions and road dust resuspension.

The content of Mn lies in the range of 10–120 ng/m^3^ with a mean value of 32.1 ± 20.9 ng/m^3^ which is ten times higher than it has been reported in New York City (Peltier & Lippmann, 2011) and similar as in Seoul megapolis, where the mean value is 41.13 ng/m^3^ (Kim et al., 2021) (Table 7). Spatial pattern of Mn distribution in urban air follows the ones for Fe and k_V_ (Fig. 7E), showing higher concentrations in cluster I.

The average concentration of atmospheric Pb is 25.0 ± 20.8 ng/m^−3^, which falls far below the limits of the current standard of EU (Directive 2008/50/EC) and World Health Organization (WHO, 2021) of 500 ng/m^3^ (Table 6, Fig. 7F). The content of Pb is slightly higher in traffic-exposed sites.

In the European Union in 2018, median value for Ni was 1.64 ng/m^3^ and the range was from 0.03 to 46.16 ng/m^3^ (EEA, 2024). In Kyiv, Ni demonstrates mean value of 19.8 ± 16.0 ng/m^3^ fitting WHO and EU standards (Table 6, Fig. 7G). Slightly elevated content of Ni is observed in locations of cluster II that makes a reason to assume its pedogenic origin.

Cd at AS4 and AS21 systematically exceeds 5 ng/m^3^—target value for this metal in the PM10 particulate fraction of ambient air (EEA, 2024) (Table 6, Fig. 7H). AS21 is located to the south of large concrete plant (Fig. 1). This type of industry is known to be an important emission source of heavy metals such as Cd, Cr, Cu, Pb, and Zn (Ogunkunle & Fatoba, 2014; Schuhmacher et al., 2004). In AS4, Cd is most likely sourced from railway operations and maintenance facilities, which are known to carry out a variety of industrial activities such as coal and crude oil combustion, metal coating, and nonferrous alloys processing.

The concentration of atmospheric total Cr exhibits moderate variation throughout the city ranging from 0 to 70 ng/m^3^. The mean level 16 ± 8 ng/m^3^ does not differ from values for cities of Radom (Poland) 25 ng/m^3^ (Swietlik et al., 2011) and Frankfurt-am-Main (Germany) 16.3 ng/m^3^ (Zereini et al., 2005) (Table 6, Fig. 7I).

### Links between magnetic susceptibility and heavy metals in PM collected on air filters

Principal component analysis was used to identify the major factors responsible for the variance of heavy metal concentrations and magnetic susceptibility of PM in air in locations from two clusters. Standardized variables were obtained by varimax rotation method.

**Fig. 11:**
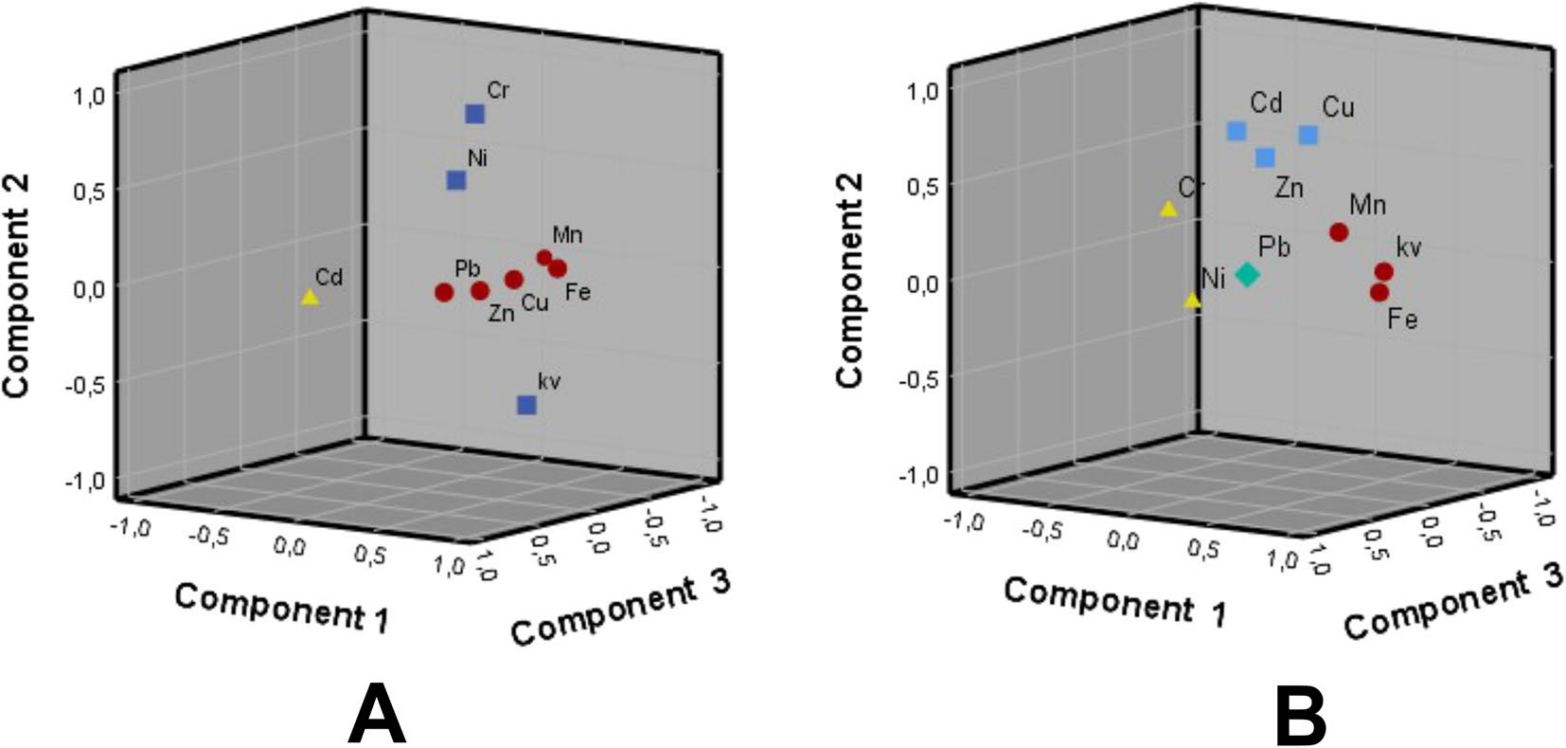
PCA results for the PM collected in AS of cluster I (**A**) and cluster II (**B**): 3D representation of loadings of the principal components (red circles, PC1; blue squares, PC2, yellow triangles, PC3; green rhomb, PC4)

Results for cluster 1 indicate that three PC with eigenvalues higher than 1 account for 63.469% of the overall variance (Table 8, Fig. 11A). PC 1 has high loadings for Fe, Mn, Zn, Cu, Pb, and k_V_, explaining 32.486% of the variance and representing road dust containing a mixture of industrial and vehicular emissions, among which prevails non-exhaust traffic contribution, such as brake and tire wear (McKenzie et al., 2009). PC 2 positively correlates to Cr and Ni and negatively to k_V_, accounting for 16.758% of the total variance that may refer to geogenic contribution, and PC 3 contains only Cd and explains 14.225% of variance is related to concrete plant and railway facilities emissions.

**Table 8:** Components extracted by PCA factorization of Cluster I air filters data characterized by their significant correlations with variables (KMO value of 0.705 and Bartlett's test < 0.001)

	PC1 Eigenvalue 3.121 Explained variability 32.486%	PC2 Eigenvalue 1.411 Explained variability 16.758%	PC3 Eigenvalue 1.180 Explained variability 14.225%	Communality
Fe	0.829			0.704
Mn	0.797			0.668
Cu	0.618			0.386
Pb	0.496			0.473
Zn	0.662			0.594
k_V_	0.545	− 0.641		0.745
Ni		0.580		0.590
Cr		0.842		0.794
Cd			0.868	0.758

Results obtained for the cluster II indicate that four PC with eigenvalues higher than 1 account for 63.997% of the overall variance (Table 9, Fig. 11B). PC 1 is correlating positively with Fe, k_V_, and Mn, and explaining 21.022% of the variance. This component represents airborne re-suspended road dust. PC 2 has high loadings to Cd, Cu, and Zn accounting for 18.578% of the total variance and representing input of various industrial emissions. PC 3 represents natural soil signal and correlates with Ni and Cr, accounting for 12.699% of variance. PC 4 contains Pb with 11.698% of the explained total variance. This component could be allocated to residual Pb in fuel, engine lubricants, and parts, as well as tire and brake wear or exhaust traffic emissions of which Pb is a source marker (Chen et al., 2022).

**Table 9:** Components extracted by PCA factorization of Cluster II air filters data characterized by their significant correlations with variables (KMO value of 0.654 and Bartlett’s test < 0.001)

	PC1 Eigenvalue 2.296 Explained variability 21.022%	PC2 Eigenvalue 1.418 Explained variability 18.578%	PC3 Eigenvalue 1.042 Explained variability 12.699%	PC4 Eigenvalue 1.004 Explained variability 11.698%	Communality
Fe	0.816				0.667
k_V_	0.796				0.644
Mn	0.677				0.581
Cd		0.753			0.628
Cu		0.668			0.598
Zn		0.651			0.548
Ni			0.799		0.692
Cr			0.514		0.468
Pb				0.966	0.934

### On the estimation of the thermal power stations emissions to urban air

Fly ashes emitted from coal-burning power plants are well-known to cause magnetic enhancement in soil and dust (Petrovsky and Ellwood, 1999; Szuszkiewicz et al., 2015). Spherical particles populated with coarse-grained magnetite of the size between 2 and 200 µm are reported to be formed during coal combustion (Evans & Heller, 2003; Strzyszcz & Magiera, 1998). During cold period of the year, fossil fuel burning power plants enhance production and the magnetic enhancement in PM is also expected. However, in Kyiv, we observe the opposite tendency of low k_V_ during winter months. To estimate a relative input of TPS into magnetic signal of PM collected in air filters, we attracted the data from the AS located in the vicinity of Trypilska TPS.

**Fig. 12:**
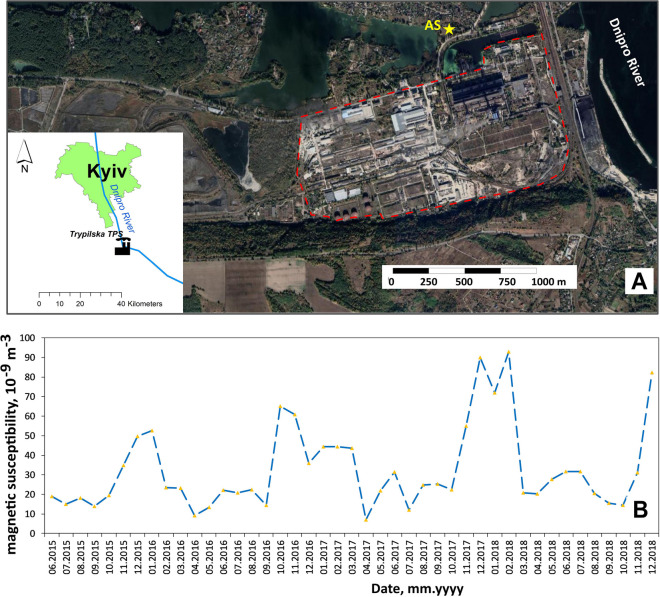
Situation map of Trypilska Thermal Power Station (**A**) and the temporal variation of magnetic susceptibility k_V_ at air sampling site nearby (**B**)

Trypilska TPS (N 50.13°; E 30.74°) is located on the Dnieper coast, 45 km south of Kyiv (Fig. 12A). It has an installed capacity of 1800 MW being the largest power generating facility in the Kyiv region. Other sources of electricity in the region are power plants TPS-5 and TPS-6 with a capacity of 700 MW and 500 MW, respectively, and Darnytska TPS with an installed capacity of 160 MW, located in the city of Kyiv (Volchin et al., 2013). The main fuel at Trypilska TPS is Donbas anthracite coal; heavy fractions of crude oil are also used.

The air sampling site is located 0.4 km to the north from the TPS at the rural area. The chemical composition of the aerosol pumped through the air filters is totally controlled by the type and quality of fuel used on TPS. So, for example, in the winter of 2016–2017, increased concentrations of chromium up to 40–60 ng/m^3^ were observed in air when the station used low-grade coal from Donbas particularly enriched with Cr (Kozij & Ishkov, 2018).

In the temporal variations of monthly k_V_ at this AS, maxima are observed in the heating season of the year which usually starts in October and lasts till March (Fig. 12B). The magnetic enhancement makes 30–60 × 10^−9^ m^−3^.

Returning to the issue of TPS contribution to magnetic PM flux in Kyiv, we can expect much weaker impact of power stations on air quality due to three reasons. Firstly, several times lower magnetic enhancement in the near vicinity of TSP in Kyiv is assumed as the capacity of Darnytsia TSP is ten times lower and TPS-5 and TPS-6 are 2.5 and 3.5 times lower in comparison to Trypilska TPS, respectively. Secondly, according to the generalized representation of decrease of magnetic deposition with distance from the source collated by Flanders (1994), at a distance of 1 km, the magnetic dust flux decreases ten times. Thus, the impact of TPS-5 will be negligible even at the nearest air sampling sites AS8 and AS20, located at 4 km distance, as well as impact of Darnytsia TSP on dust flux at AS9 and AS4, located at more than 2 km from TSP. Thirdly, TPS in Kyiv use crude oil and natural gas rather than coal as fuel thus releasing far less particulate matter emissions in the atmosphere (Finkelman & Greb, 2008). In addition, particles released due to fuel combustion are those with lower magnetic concentration compared to coarse ones originating from tire and brake abrasion and accumulated in street dust (Revuelta et al., 2014; Jordanova et al., 2023) .

## Conclusions and recommendations

In the current study, the topsoil and tree bark magnetic susceptibility maps of Kyiv megapolis are analyzed together with particulate matter examined through magnetic susceptibility and chemical measurements on air filters. The magnetic and heavy metal composition data for the topsoil provide a historical perspective of industrial pollution over the last century, revealing areas affected by industries that have since closed or changed their production profiles. The tree bark susceptibility map reflects the situation chronologically limited to the last 2–3 decades (the average age of sampled trees), thus showing cumulative effect of particulate matter emissions. The patterns of spatial distribution of magnetic susceptibility of topsoil and tree bark demonstrate great similarity, confirming that fossil fuel burning power plants, dusting ash dumps, railway facilities, metalworking, and machinery-building factories shape the composition of particulate falling on the ground in urban area, with the exception of near-road areas of street and squares with heavy traffic.

Enhanced concentrations of Pb, Cu, and Zn were observed in urban soils. Magnetic susceptibility showed a significant positive correlation with Ni, Cu, and Zn and with pollution load index as well, supporting the use of topsoil as a tool for mapping anthropogenic pollution in Kyiv. Source apportionment analysis suggests that Mn and Ni originate primarily from natural soil, while other metals are likely sourced from anthropogenic activities, such as vehicular emissions, fossil fuel combustion, and industrial processes.

The impact of anthropogenic activities on air quality during the years 2015–2018 was characterized by analyzing the quantity, distribution, and composition of heavy metals and magnetic susceptibility in particulate matter collected in air filters. The obtained results strongly depend on the location of air samplers. The road dust, re-suspended by moving vehicles, dominated temporal variation of PM magnetic susceptibility that is particularly visible at the streets with heavy traffic. The air at busy streets was cleaner in winter, when the street dust is immobilized by snow cover, frozen, or bound in wet soil. During warmer months, the magnetic susceptibility and concentrations of associated heavy metals (Fe, Mn, Cu, Zn) increased by two to four times. The thermal power stations in Kyiv pose no significant effect on air quality on air sampling sites due to relatively distant location and limited use of coal as fuel. The concentrations of Pb, Cu, Zn, Cr, Cd, and Ni are at normal urban level with the exception of the site directly exposed to industrial input. In areas with heavy traffic, the air is enriched with Fe and Mn, comparable to levels found in heavily industrialized regions worldwide.

Principal component analysis (PCA) revealed two distinct patterns of air pollution regarding magnetic particles and heavy metals. The first one is relevant to the streets with heavy traffic, originating mainly from street dust re-suspension by vehicles. The second pattern is recognized in residential yard areas, where due to lower volumes of airborne street dust, contribution of industrial and exhaust vehicular emissions can be distinguished.

Based on the results obtained, we make recommendations for mitigation of risks from soil and air pollution with heavy metals in Kyiv. The implementation of measures to control the amount of street dust is crucial. Regular street cleaning and utilizing dust suppressants particularly in high-traffic areas or close to industrial zones will help to remove accumulated dust and prevent it from becoming airborne. Incorporating policies for cleaner transport and imposing restrictions on dirty vehicles is also recommended. In order to avoid the remobilization of heavy metals and other harmful substances, the soil cover at the sites of old factories may be replaced.

The existing program for ecological monitoring in Kyiv must be enriched with magnetic measurements on soils and PM collected by low-volume dust samplers (Górka-Kostrubiec et al., 2024). Topsoil magnetic susceptibility mapping using soils and biomonitors as the geographic-based tool should be implemented in environmental risk assessment and forecasting methodologies that enable the decision-makers to anticipate the type of ecological control necessary in the city.

The integration of long-term and short-term information on anthropogenic pollution originating from magnetic method will help to identify first-to-be-done measures to sustain the environmental quality and carrying capacity of Kyiv, currently experiencing deep socio-economic upheaval due to the ongoing war.

## Data Availability

No datasets were generated or analysed during the current study.
