# Correction to: Long‑ and short‑term pollution effect in megapolis assessed from magnetic and geochemical measurements on soils, tree trunk bark, and air filters

**DOI:** 10.1007/s10661-025-14143-x

**Published:** 2025-06-02

**Authors:** Kseniia M. Bondar, Iryna V. Tsiupa

**Affiliations:** 1 Institute of Geophysics, Polish Academy of Sciences, Ksiecia Janusza 64, 01‑452, Warsaw, Poland; 2 Taras Shevchenko National University of Kyiv, 90 Vasylkivska Str, Kiev, 03022 Ukraine

**Correction to** **:** **Environ Monit Assess (2024) 196:1041**

10.1007/s10661-024-13194-w

The original version of this article unfortunately contained an error in Table 7.

Table 7 has been corrected as follows.

Wrong Table 7:

**Table 7:** Summary on PM`s magnetic susceptibility k_V_(10^-9^ m^-3^) and HM contents (ng m^-3^) in urban air

	Dataset for entire city $(n=294)$		Cluster I (AS7, AS9, AS20) Heavy traffic area (n=129)		Cluster II (AS4, AS8, AS21) Yard residential area (n=122)	
	Range	Range	Range	Range	Range	Mean±SD
${k}_{v}$	3.02–211	14.4–100	3.02–81.1	3.02–81.1	14.4–100	49.0±19.2
Pb	0–220	0–220	0–120	0–120	0–220	26.4±24.0
Cd	0–80	0–20	0–80	0–80	0–20	3.13±4.57
Mn	10–120	10–120	10–70	10–70	10–120	34.6±17.9
Ni	0–200	0–70	0–200	0–200	0–70	18.6±13.1
Cr	0–70	0–70	0–70	0–70	0–70	15.7±11.2
Fe	60–5170	60–5170	200–2520	200–2520	60–5170	1226±772
Cu	0–360	0–310	10–360	10–360	0–310	60.0±34.9
Zn	0–770	0–320	10–480	10–480	0–320	90.0±48.3

Correct Table 7:

**Table 7:** Summary on PM`s magnetic susceptibility k_V_(10^-9^ m^-3^) and HM contents (ng m^-3^) in urban air

	Dataset for entire city (n=294)		Cluster I (AS7, AS9, AS20) Heavy traffic area (n=129)		Cluster II (AS4, AS8, AS21) Yard residential area (n=122)	
	Range	Mean±SD	Range	Mean±SD	Range	Mean±SD
k_V_	3.02 - 211	49.8±37.4	14.4 – 100	49.0±19.2	3.02 – 81.1	26.5±13.6
Pb	0 - 220	25.0±20.8	0 – 220	26.4±24.0	0 – 120	23.2±18.8
Cd	0 - 80	6.72±12.3	0 – 20	3.13±4.57	0 – 80	13.4±15.96
Mn	10 - 120	32.1±20.9	10 - 120	34.6±17.9	10 – 70	21.6±10.8
Ni	0 - 200	19.8±16.0	0 – 70	18.6±13.1	0 – 200	21.5±19.5
Cr	0 - 70	15.9±10.9	0 – 70	15.7±11.2	0 – 70	16.6±10.5
Fe	60 - 5170	1156±758	60 – 5170	1226±772	200 – 2520	828±366
Cu	0 - 360	58.1±40.2	0 – 310	60.0±34.9	10 – 360	51.3±44.0
Zn	0 - 770	92.1±73.4	0 – 320	90.0±48.3	10 – 480	76.0±51.4

